# Dynamics of Quadriceps Muscles during Isometric Contractions: Velocity-Encoded Phase Contrast MRI Study

**DOI:** 10.3390/diagnostics11122280

**Published:** 2021-12-06

**Authors:** Toshiaki Oda, Vadim Malis, Taija Finni, Ryuta Kinugasa, Shantanu Sinha

**Affiliations:** 1 Life and Health Sciences, Hyogo University of Teacher Education, Kato 673-1494, Japan; 2 Muscle Imaging and Modeling Laboratory, Department of Radiology, University of California San Diego, San Diego, CA 92093, USA; vmalis@ad.ucsd.edu; 3 Neuromuscular Research Center, Faculty of Sport and Health Sciences, University of Jyväskylä, 40014 Jyväskylä, Finland; taija.m.juutinen@jyu.fi; 4 Department of Human Sciences, Kanagawa University, Yokohama 221-8686, Japan; rk@jindai.jp

**Keywords:** aponeurosis, deformation, isometric, quadriceps muscles, thigh, velocity-encoded phase contrast MRI

## Abstract

Objective: To quantify the spatial heterogeneity of displacement during voluntary isometric contraction within and between the different compartments of the quadriceps. Methods: The thigh muscles of seven subjects were imaged on an MRI scanner while performing isometric knee extensions at 40% maximal voluntary contraction. A gated velocity-encoded phase contrast MRI sequence in axial orientations yielded tissue velocity-encoded dynamic images of the four different compartments of the thigh muscles (vastus lateralis (VL), vastus medialis (VM), vastus intermedius (VI), and rectus femoris (RF)) at three longitudinal locations of the proximal–distal length: 17.5% (proximal), 50% (middle), and 77.5% (distal). The displacement, which is the time integration of the measured velocity, was calculated along the three orthogonal axes using a tracking algorithm. Results: The displacement of the muscle tissues was clearly nonuniform within each axial section as well as between the three axial locations. The ensemble average of the magnitude of the total displacement as a synthetic vector of the X, Y, and Z displacements was significantly larger in the VM at the middle location (*p* < 0.01), and in the VI at the distal location than in the other three muscles. The ensemble average of *Z*-axis displacement, which was almost aligned with the line of action, was significantly larger in VI than in the other three muscles in all three locations. Displacements of more than 20 mm were observed around the central aponeuroses, such as those between VI and the other surrounding muscles. Conclusions: These results imply that the quadriceps muscles act as one functional unit in normal force generation through the central aponeuroses despite complex behavior in each of the muscles, each of which possesses different physiological characteristics and architectures.

## 1. Introduction

The extensor muscles of the knee joint play important roles in several human movements. In dynamic and ballistic movements such as jumping in sports, these muscles are the main contributors to the acceleration of the center of gravity of the body mass [1]. In terms of clinical significance, the deterioration of the functioning of the knee extensors is significantly related to the decrease in the quality of life of the subject [2]. Standing up from a seated or prone position, as well as climbing stairs, depends critically on leg press performance. Sarcopenia and atrophy of knee extensor muscles with aging, muscular disuse, and inactivity result in severe hindrance to daily activities and reduced self-dependence. Additionally, it has been reported that some knee injuries, such as that of the anterior cruciate ligament, prohibit normal muscle function of the knee extensors [3]. Thus, these muscles are considered to play key roles in enabling normal physical functions in humans. It is, thus, important to investigate the functional features of these knee extensor muscles.

**Figure 1:**
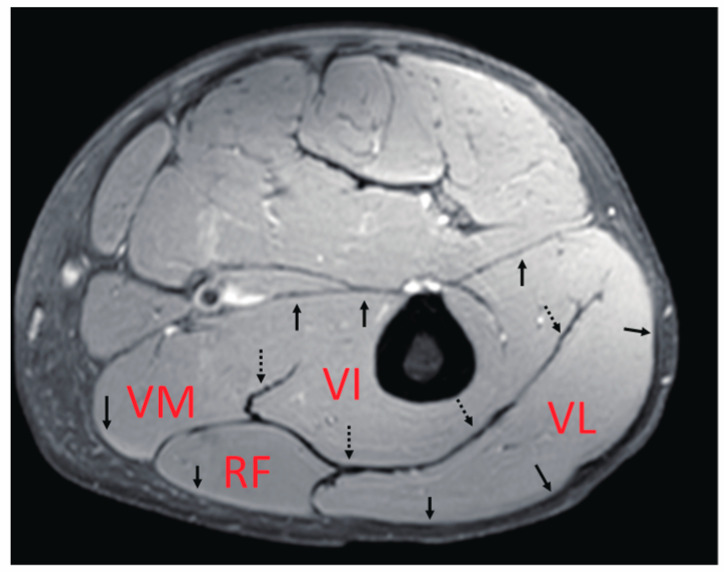
An example of the anatomical MR cross-sectional image of the center of the thigh. The quadriceps muscle includes the four heads of the muscle bellies of the vastus lateralis (VL), vastus medialis (VM), vastus intermedius (VI), and the rectus femoris (RF). The arrows show the aponeuroses surrounding the quadriceps muscle. The dotted arrows point to the thick aponeuroses referred to as “central aponeuroses” in this study.

The main knee extensor is the quadriceps muscle. As the name suggests, this muscle includes the four heads of the muscle bellies of the vastus lateralis (VL), vastus medialis (VM), vastus intermedius (VI), and rectus femoris (RF). VL, VM, and VI are mono-articular muscles crossing one joint at the knee, while RF is a bi-articular muscle operating over two joints, including the hip and knee. In terms of their locations inside the thigh, only the VI, reported to contribute significantly to the generation of knee extension torque [4], is located in a deep position near the femur, while the others are more superficial. In addition, as summarized by Finni et al. [5], their fascicle architectures as an anatomical morphology of bundles of muscle fibers [6,7], as well as their fiber compositions [8,9], are quite complicated compared with those of the medial gastrocnemius, which has been thoroughly investigated. Thus, it is likely that these variations in the characteristics of each muscle generate differences in the contraction behavior of deformation, such as length change, thereby, significantly affecting the force generation function of these muscles. Furthermore, quadriceps muscles have thick well-developed common aponeuroses between muscles (Figure 1), within which force and deformation may be coupled, giving rise to the possibility of synchronized length changes at these junctions. However, detailed knowledge of the muscle deformation of separate quadriceps muscles during contraction is limited.

Using dynamic MR imaging with gated velocity-encoded phase contrast MRI (VEPC-MRI), the mechanics of both muscle and aponeuroses have been reported [10,11]. Csapo et al. [12] demonstrated that, in calf muscles, the deformation of local muscle tissues is positively correlated with the local muscle fiber activation measured by electromyography (EMG). Additionally, they observed considerable heterogeneity in the spatial distribution of displacement and/or activation both in the longitudinal (i.e., the proximal–distal direction) and transverse directions during the contraction cycle. The observed heterogeneity in the activation of calf muscles can be attributed to the combination of architectural and physiological differences in muscle fiber behavior among calf muscles, resulting in the functional heterogeneity of the contracting muscles. Using similar methods, Finni et al. [5] investigated the dynamics of thigh muscles during contractions and demonstrated the changes in the outline shape and aponeuroses behavior of muscles, but not in the magnitude and displacement direction of the individual muscles and aponeuroses at different longitudinal locations.

The hypothesis of this study is that, at a given level of voluntary force production, the displacements of the four muscles in the quadriceps are different (heterogeneous) both over the axial cross-section and along the longitudinal direction. However, they synchronize in terms of their displacements near the common aponeuroses. These behaviors can reflect the functional differences between the four muscles. To capture this aspect, deformations within the entire thigh muscles were mapped. To elucidate the behavior of the quadriceps muscles during contractions, gated VEPC-MR images were acquired with a specially designed MR-compatible torque measurement apparatus, yielding velocity measurements at each anatomical point at different time phases during the contraction cycle. Subsequently, tracking algorithms were applied at three different cross-sectional slices along the longitudinal axis, yielding the spatial distribution of the displacement.

## 2. Methods

### 2.1. Subjects

Seven healthy male subjects (age: 27.7 ± 7.7 years, height: 170.1 ± 3.1 cm, weight: 66.2 ± 7.3 kg, thigh length: 40.4 ± 1.9 cm) were chosen for the experiments after obtaining their consent as approved by the Institutional Review Board of University of California, San Diego. Exclusion criteria included any prior injury such as ACL tears, and the usual exclusion criteria for MRI examinations, such as the presence of cardiac pacemakers and metallic imbedded devices. All subjects provided written informed consent to participate in the study, which was performed in accordance with the guidelines of the Declaration of Helsinki.

### 2.2. MRI Compatible Thigh Muscles Isometric Contractions Imaging Device

**Figure 2:**
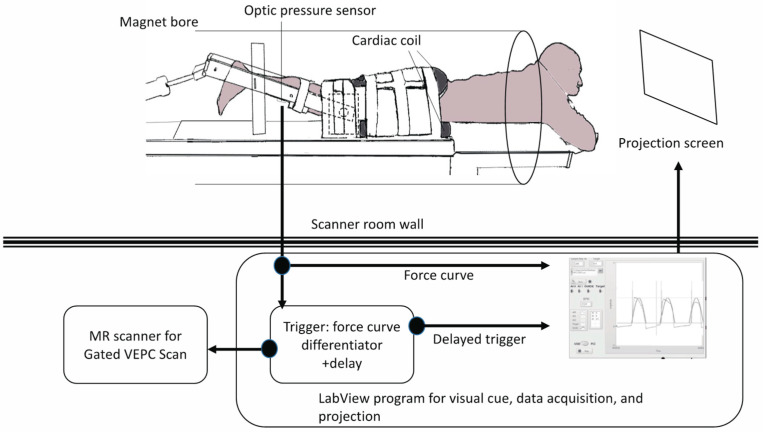
Illustration of the experimental setup of velocity-encoded phase contrast (VEPC) image acquisition.

A previously reported device for imaging isometric, eccentric, and concentric contractions of the lower leg [11,12] was modified to image the thigh muscles (Figure 2). The subject lay prone and leg first in a 1.5T MRI scanner (Signa HDx, GE Medical Systems, Chicago, IL, USA), with the thigh horizontal inside the anterior and posterior parts of an eight-channel cardiac coil. To ensure that the thighs did not move upward during the downward exertion of the tibia (see below), the thighs were stabilized by Velcro restrainers over the gluteal muscles, cardiac coils, and at the knee. The tibia rested at a pre-determined angle (35° knee flexion) on a firmly anchored carbon fiber plate, which had an optical force transducer super-glued on the opposite side. It could measure the amount of force exerted by the subject through the tibia. The output of the force transducer was converted to an analog signal using a spectrophotometer (Fiber Scan, Luna Innovations, Roanoke, VA, USA), digitized using an analog-to-digital converter (ADC), and then inputted to a computer and used for various purposes, including being recorded for subsequent analysis. An indigenous algorithm developed for this study using LabView (National Instruments, Austin, TX, USA) and MATLAB (The MathWorks, Natick, MA, USA) synchronized the time period of the contractions to 3 s in these experiments. The subject was trained to follow a visual target guide generated by the same algorithm on the computer and projected onto a screen at the base of the patient gurney table (the patient could view this while being scanned) while exerting isometric contractions. The accuracy and consistency of the force exerted over different cycles were enhanced by a biofeedback system in which the force exerted, sensed by the force transducer, was projected back through the same computer algorithm as an overlay on the target visual guide. The subject was instructed to follow the guide and correct the timing and magnitude of force. The indigenous algorithm also differentiated the force output to generate a trigger output that was used to trigger the gated scan on the MRI scanner. Before the start of the MRI scans, three trials of the subjects exerting maximum voluntary contractions (MVC) were recorded. The target level of the force was then adjusted to a percentage of the MVC value for the subject to exert during subsequent MR acquisition. The force level was 40% of MVC.

### 2.3. MR Acquisitions

In this study, isometric contractions were studied. In this experimental condition, although the joint was fixed, internal shortening of muscle fascicles could arise owing to the viscoelastic mechanical properties of the connective tissues, including aponeuroses and tendons [13]. In the quadriceps muscles, the complex radial architecture of fascicles made it difficult to obtain the change in displacement in muscle tissues from the sagittal plane of the MR images compared with the gastrocnemius muscles [12], which have been well investigated in previous studies. In addition, a comparison of the displacements of the four muscle tissues at the same time was important. Thus, we selected the cross-sectional plane as the measurement plane.

The protocol used in these experiments was the same as that used and tested for reliability in our laboratory in the past [12]. Three axial slices were chosen in the middle of the thigh using a three-plane localizer on the sagittal scout plane, and at 17.5% proximal and distal to the mid-point. Subsequently, axial VEPC images were acquired in each of the three locations (i.e., proximal, middle, and distal) using a gated FAST-GRE sequence. The scans were velocity-encoded in all three directions to 10 cm/s, with an echo time/repetition time of 6.3/24.2 ms, two excitations, 20° flip angle, 256 × 256 matrix, 5 mm slice thickness, and 20 × 14 cm field of view gated with 22 phases per cycle of 3 s. The voxel sizes in the X, Y, and Z directions were 0.78 mm, 0.55 mm, and 5 mm, respectively. With four views per segment, this resulted in a typical total of 70 contractions to be exerted by the subject for VEPC image acquisition.

### 2.4. MRI Post-Processing and Image Analyses

The post-processing procedure was similar to that described by Csapo et al. [12], who reported the muscle behavior of the triceps surae muscles using in-plane VEPC-MRI. The validity and reproducibility of the displacement tracking used in this study are reported elsewhere [11,14,15].

The velocity-encoded images were first corrected for shading artifacts arising from magnetic field inhomogeneities and chemical shifts [11]. Then, as the velocity-encoded images were sensitive to noise, an anisotropic diffusion filter was applied to the velocity images similar to that applied by Sinha et al. [16]. The anisotropic diffusion filter reduces noise and slows the velocity components in homogeneous regions while preserving the edges, thus preserving the effective resolution of the velocity images. The anisotropic diffusion filter function in the image processing toolbox of MATLAB (MathWorks) was used in this study. As input parameters in this function, low values of *K* (=2) and several iterations (=10) were selected, as the phase contrast images did not have strong edge content.

Subsequently, the displacement of each voxel of the muscle tissue was tracked on a frame-by-frame basis throughout the contraction–relaxation cycle for each muscle in all planes. For this purpose, the images reflecting the velocity in the X- (medial–lateral) and Y-directions (anterior–posterior) in the VEPC images were evaluated to track the in-plane displacement of each volume element. Accounting for the third velocity component (proximal–distal), the magnitude of the total displacement with respect to the original position in the first frame was then calculated as the square root of the sum of squared displacements in each direction (DISP = $\sqrt{X^{2}+Y^{2}+Z^{2}}$). It may be noted that the through-plane tissue movements (Z direction) could not be tracked owing to the 2D nature of slice acquisition. Therefore, the uniformity of the velocities in this axis in a given phase had to be assumed in a manner similar to that of Csapo et al. [12]. Specifically, after displacement tracking of X and Y in each phase, the velocity in the Z direction at the destination voxel of X and Y tracking was used in the next phase for time integration and added to the *Z*-axis displacement. This assumption was based on the high correlation coefficient (R > 0.98) between the Z displacement estimated by this method and the measured Z displacement and the slight systematic error (less than 1 mm) in the quadriceps muscle during submaximal contraction, as reported by Finni et al. [5].

Then, to prevent the high velocity data observed from voxels on blood vessels from interfering with the data of muscle tissue movement, the voxels corresponding to the vessels were identified algorithmically by considering if the displacements return to their original locations on the completion of a cycle. This was because the vessel regions were characterized by a consistent increase or decrease in tissue displacement during the contraction phase.

Moreover, although the thigh was tightly fixed to the experimental device by straps, thigh movements still occurred in X–Y plane because of the stresses produced between the torque measurement part of the machine and the restraining straps whenever force was exerted. However, there was little movement in the Z direction since the knee position was firmly fixed by the experimental device, while the anterior surfaces of the thighs were in contact with the surface of MRI (patient) bed. To minimize this extra error of the X–Y displacement from the cross-sectional images of VEPC, the circumference of the femoral marrow (appearing very bright in the MR image) was traced manually by an anatomical specialist from the anatomical images (using ImageJ, NIH) and digitally masked. The displacement of the position of its center of gravity was then quantified in all the 22 phases. The displacement of the femoral marrow in each phase was subtracted from the tracked displacement of the muscle tissue in that particular phase. Typically, the maximal value of the medial to the lateral direction was 2.7 ± 1.8 mm, while that in the anterior to posterior direction was 8.9 ± 4.8 mm, from relaxation to contraction through the 22 phases. This correction process resulted in a good agreement between the calculated results and the measurements of the movement in the anatomical image for the muscle tissue near the intersection with the aponeuroses.

### 2.5. Total Displacement

The magnitude of the synthetic vector of the X, Y, and Z displacements was calculated and is shown as the total displacement. In the temporal course of the change in displacement, the total displacement of each voxel was defined as the difference between the smallest and largest values through contraction. The average value of each muscle belly in each slice was used for statistical comparison.

### 2.6. Z-Axis Displacement

The displacement along the *Z*-axis demonstrated the deformation of muscle tissues in the proximal–distal direction, while the X and Y axes showed the medial–lateral and anterior–posterior directions, respectively. One of the most important functions of the muscle–tendon complex of the knee extensors is to pull the patellar tendon in the Z direction (approximately co-linear with the line of action) to generate mechanical work and joint torque for the extension of the knee joint. The *Z*-axis displacement was defined as the displacement from the smallest to the largest values through contraction. The average value of each muscle belly in each slice was used for statistical comparison.

### 2.7. Ratio of the Z-Component Displacement Vector to the Total Displacement during Contraction

To evaluate the dominant direction of tissue displacement between the proximal and distal, medial–lateral, or anterior–posterior directions, the ratio of the Z-component displacement to the total displacement was calculated for all voxels and ranged from −1 to 1. If this ratio was near 1 or −1, it meant that the displacement along the proximal–distal (Z) direction was dominant among the three directions.

### 2.8. Comparison of the Average Displacement between the Muscles

The magnitude images in the resting condition in the images generated by VEPC sequence were used to outline the contours of the VL, RF, VI, and VM muscles. In the proximal portion, since the VM had not yet appeared, only the other muscles were outlined. The average value for each muscle was calculated.

### 2.9. Statistics

The data are reported as mean ± SD. Statistical analyses were performed using IBM SPSS Statistics 22 (IBM, Endicott, NY, USA). Repeated-measures of ANOVA were used to detect significant displacement effects. This analysis was performed between the muscles in each portion (e.g., VL vs. RF vs. VI vs.VM in the proximal portion) and between the positions of one muscle (e.g., proximal vs. middle vs. distal in VL muscle). If the F-statistic of the ANOVA was significant, differences between means were assessed using Tukey’s HSD post-hoc test. In the proximal portion, as the muscle belly of the VM was not visible in the imaging plane, a comparison only between VL, RF, and VI was performed. The level of statistical significance was set at *p* < 0.05.

## 3. Results

**Figure 3:**
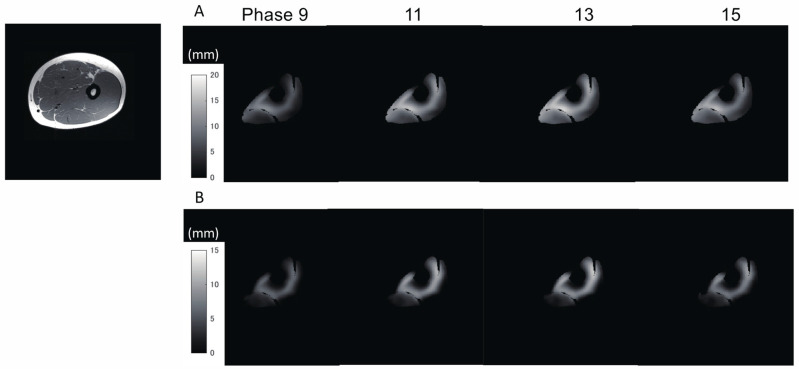
A typical example of the temporal changes in total (**A**) and Z-axis (**B**) displacements in one subject at phase 9, 11, 13, and 15 (the force onset was at phase 9 and the peak force was generated in phase 13). The gray scale represents the distribution of the magnitude of displacement.

The peak forces generated voluntarily during the measurements in the three sections of the thigh were 39.5 ± 1.5% in the proximal section, 39.4 ± 1.7% in the middle section, and 38.8 ± 2.5% in the distal section, and they did not exhibit statistically significant differences. Furthermore, the low coefficient of variation for each task (3.7%, 4.3%, and 6.4%, respectively) showed stable reproducibility of the generation of all forces in the series of repeated contractions. Figure 3 shows typical examples of the displacement distribution over time. With a gradual increase in force, the displacement of the muscle tissues tended to increase.

**Figure 4:**
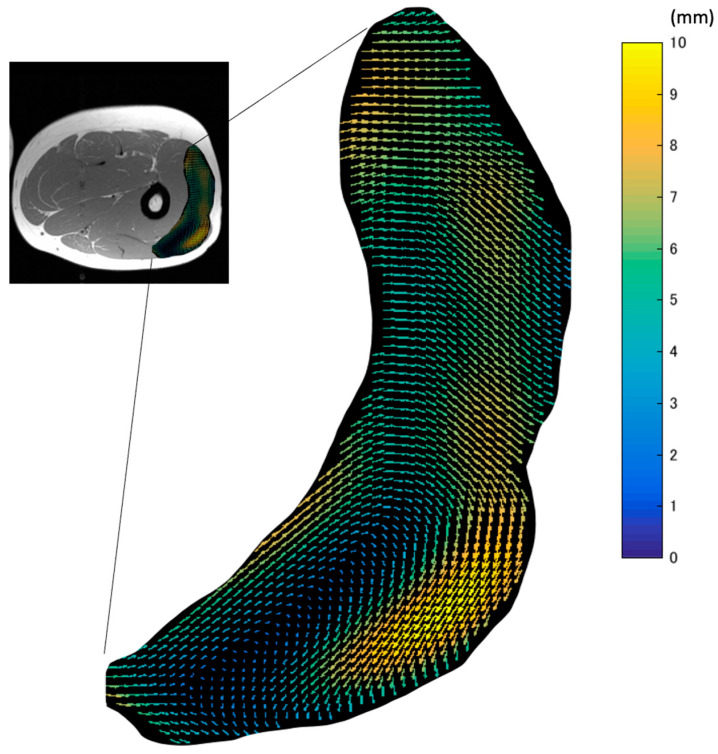
A map of the vectors of the in-plane maximal displacement of VL from the relax phase. The length and color of arrows in VL show the magnitudes of the vectors.

A typical example of the in-plane displacement (Figure 4) from rest, calculated from tracking muscle tissues in LG, demonstrated the distribution of heterogeneous displacement even within the same muscle. Both the magnitude and the direction of displacement showed considerable variation.

### 3.1. Comparison of the Total Displacement between Muscles

**Figure 5:**
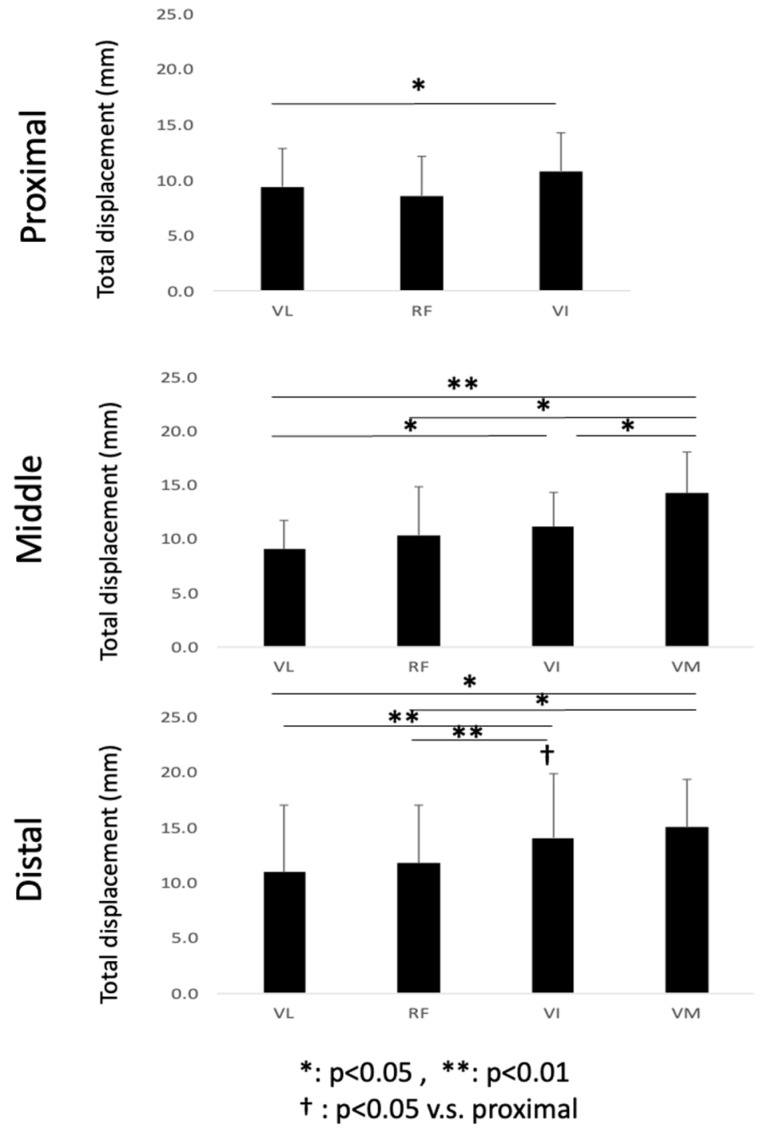
Ensemble mean value of the total displacement of the three sections, proximal, middle, and distal, for the different muscles, VL, RF, VI, and VM.

The ensemble mean value of the calculated total displacement is shown in Figure 5 for the proximal, middle, and distal sections of the different muscles, VL, RF, VI, and VM. A significant difference is observed between the VL and VM when comparing different muscles in the proximal section (*p* = 0.045). In the middle section, significant differences are observed between the VL and VI (*p* = 0.02), and the total displacement of VM is observed to be greater than others by 28–57% (*p* = 0.01; VL, *p* = 0.04; RF, *p* = 0.01). In the distal section, the total displacement of VI is significantly greater than that of VL and RF (both *p* = 0.01). The total displacement of the VM is greater than that of both the VL and RF (*p* = 0.03, *p* = 0.04, respectively). In the comparison of the same muscles in different longitudinal planes, the total displacement of only the VI in the distal section is greater than that in the proximal section (*p* = 0.02).

### 3.2. Comparison of Z-Axis Displacement between Muscles

**Figure 6:**
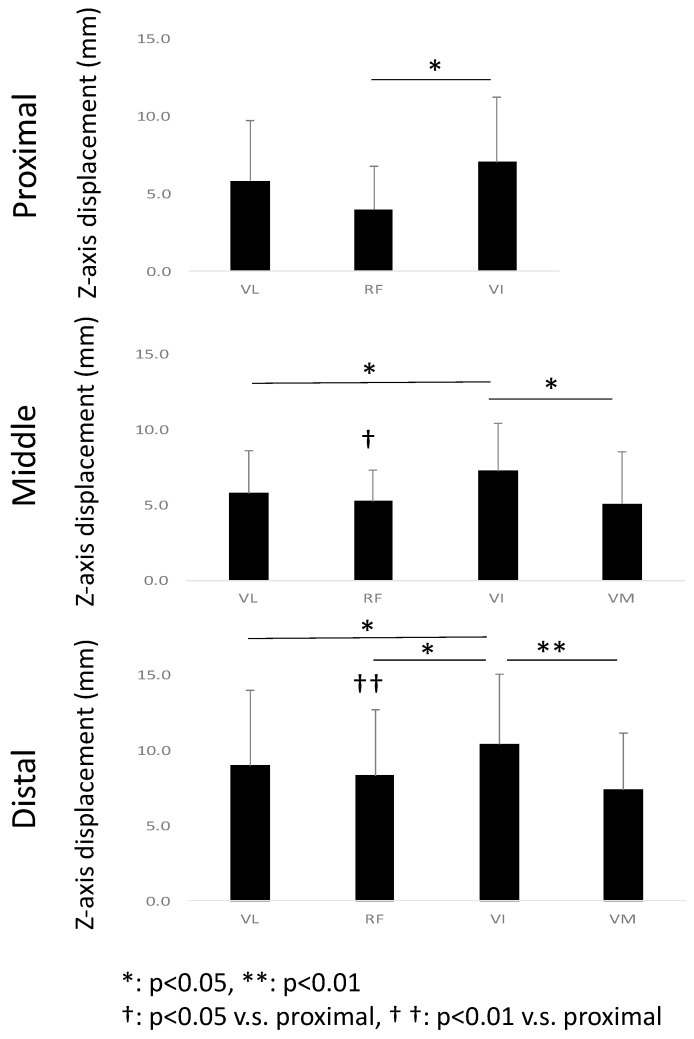
Ensemble mean value of the *Z*-axis displacement of the three sections, proximal, middle, and distal, for the different muscles, VL, RF, VI, and VM.

Figure 6 shows the ensemble mean value of the variation in the *Z*-axis displacement. In the proximal section, VI shows a statistically significantly larger *Z*-axis displacement than in RF (*p* = 0.01). In the middle section, VI shows a statistically larger *Z*-axis displacement than the VM (*p* = 0.01). In the distal section, the *Z*-axis displacement in VI is larger than that in VL (*p* = 0.01) and VM (*p* = 0.01). VI shows a larger *Z*-axis displacement than all the other muscles in all sections. The comparison of the muscles between the planes revealed that the *Z*-axis displacement in the RF was larger in the distal section than in the proximal section (*p* = 0.01).

### 3.3. In-Plane Distribution of the Total Displacement and That along the Z-Axis

**Figure 7:**
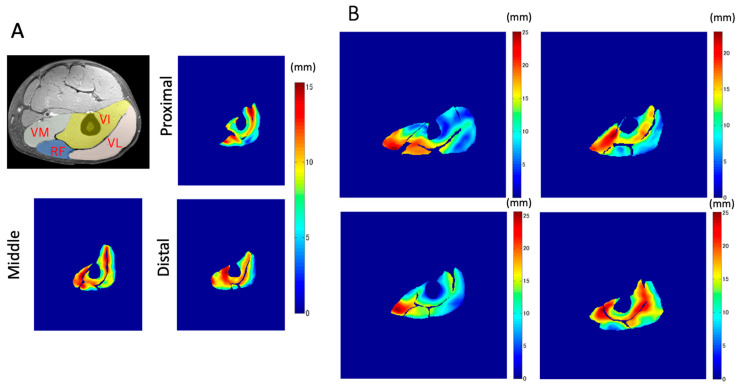
Examples of the maximal displacement at voxel level superimposed upon the static anatomical shape. (**A**) The distribution in the three different sections, proximal, middle, and distal in the same subject. (**B**) Examples of the middle section in four different subjects.

Examples of the maximal displacement at the pixel level superimposed on the static anatomical images are shown in Figure 7. The distribution of the displacements shows a remarkably large variation, even within each muscle in each plane. Large total displacements of over 20 mm are observed around the aponeuroses, such as those between VI and other muscles, and in VM and RF.

**Figure 8:**
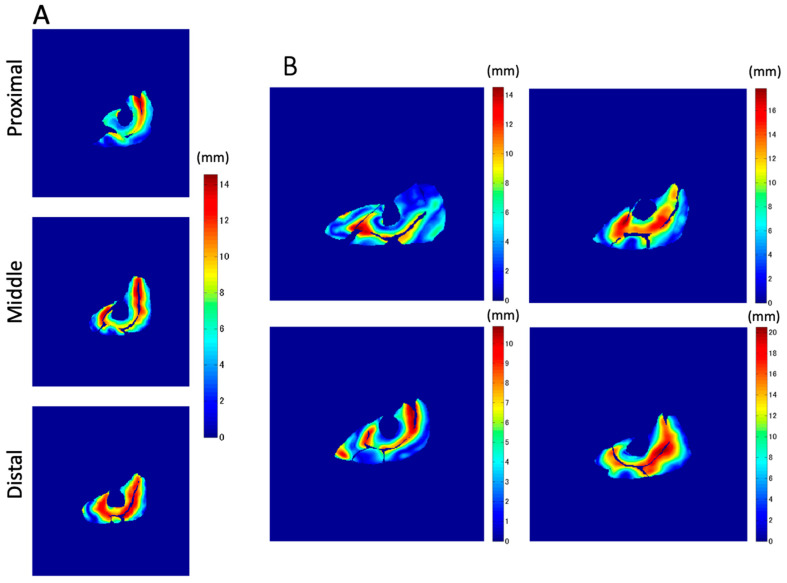
Examples of the *Z*-axis displacement at pixel level superimposed upon the static anatomical shape. (**A**) The distribution in the three different sections, proximal, middle, and distal in the same subject. (**B**) Examples of the middle section in four different subjects that clearly show all four muscles.

Figure 8 shows similar distributions of the *Z*-axis displacement in each plane in a similar set of sections and subjects as shown in Figure 7. Once again, the highest values of *Z*-axis displacement tend to concentrate around the aponeurosis (in particular, central aponeurosis) separating the VI from its surrounding muscles. The amplitudes of the displacements of the muscle tissues on one side of the central aponeurosis demonstrate similar values to those in the opposite direction. The area with a high value of *Z*-axis displacement (displaced in red) is smaller than that in the case of total displacement. However, the displacement of the muscle tissues, further away from the central aponeurosis, is smaller than that near the central aponeuroses and shown in blue.

### 3.4. Ratio of Z-Component Displacement Vector to the Total Displacement

**Figure 9:**
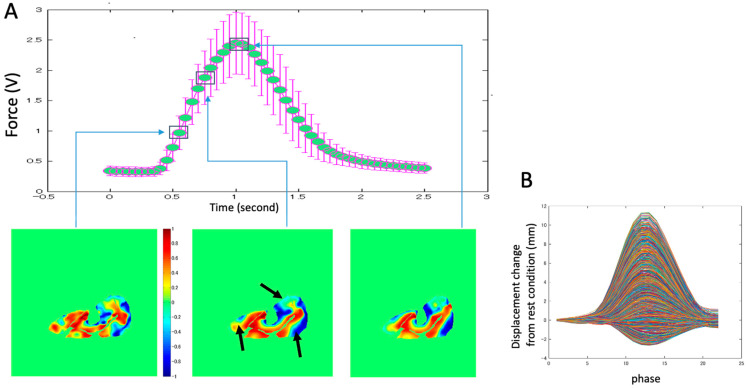
(**A**) Example of temporal variation of the ratio of the Z-component displacement vector to the total displacement. The bottom figures demonstrate the distribution of the ratio of the *Z*-axis displacement and the total displacement of the three phases during contraction. The values near 1 or −1 imply that the *Z*-axis (out-of-plane) displacement is larger than the in-plane displacement. The positive values are from the distal to the proximal, and the negative values are from the proximal to the distal. (**B**) The temporal variation of the distribution change in the Z-component displacement vector for all voxels in the same trial is shown in Figure 9A. Most pixels demonstrate a positive increment and decrement, while some pixels show small negative values.

Examples of the time variation of the ratio of the Z-component displacement vector to the total displacement in the contraction phase are shown in Figure 9A. The ratio around the central aponeurosis consistently exceeds 0.5. The other portion, indicated by arrows in the bottom figures of Figure 9A, shows a smaller value of less than 0.4. However, areas such as those near the surface boundary in the VL and near the bone in the VI muscles demonstrate negative values close to −1. In fact, through the course of contraction over time, most pixels in the bottom figures show positive displacement changes, although some have negative changes (Figure 9B). Although the details of the distribution of the ratio varied among subjects, the tendency mentioned above was common in all subjects.

## 4. Discussion

To the best of our knowledge, this is the first study that has focused on the spatial distribution of the displacement of muscle tissues and their differences in the synergist muscles in the quadriceps muscles using VEPC-MRI during voluntary isometric contraction. The main findings of this study are as follows: (1) Both the total and *Z*-axis displacements within the same cross-section differed among the different muscles comprising the quadriceps muscles. The total displacements of VI and VM, and the *Z*-axis displacement of VI, tended to be larger than that of the other muscles. (2) Both the total displacement of the VI and the *Z*-axis displacement of the RF were larger in the distal section than in the same muscles in the other two longitudinal sections (middle and proximal). (3) The largest displacements were concentrated around the central aponeuroses separating the VI from its surrounding muscles, with the *Z*-axis displacements showing the strongest tendency.

From the results of the comparison of the mean values of displacements, the heterogeneity in displacement among the four synergist muscles was observed in both the total and *Z*-axis displacements. It should be noted that the main role of the muscle–tendon complex in generating physical movements is to generate not only the tension force along the direction of the line of action, but also mechanical work. Since the mechanical work is determined as the product of force and displacement, it is important to experimentally measure the displacement of the muscle tissues along the *Z*-axis, corresponding approximately to the line of action. Thus, the 57% larger *Z*-axis displacement of VI suggests that its contribution to mechanical work is higher than that of the other muscles in the quadriceps. This, in turn, suggests that the VI is the main generator of force and mechanical work, at least in terms of displacement among the four muscles. Furthermore, a tendency of larger displacements in the distal compared to the proximal regions was observed in VI (83% higher). Other muscles showed similar behavior with larger displacement in the distal compared to the proximal, although such differences were not statistically significant (e.g., *p* = 0.07 for both proximal and distal regions for VM and VL). This result showed that, even in one muscle belly, the proximal tissue was not deformed significantly, whereas the distal tissue was extensively deformed. This might have resulted from the high contribution of the distal component to force and work (displacement) generation along the muscle belly.

Zhang et al. [4] estimated the contribution of each muscle to the knee extension torque in the quadriceps during isometric contractions using combinations of induced contraction by electrical stimulation and voluntary submaximal contractions. The contribution was reported to be the highest in VI (39.6–51.8%), while the contribution of VL and VM increased with an increase in the joint torque. The contribution of VI was shown to be high using EMG. However, in the present study, the high contribution of VI was shown in terms of displacement and mechanical work.

The nonuniform distribution of displacement observed in the present study may be linked to the distribution and orientation of the forces generated by the muscle fibers [17]. In terms of the VI, longer fibers were found in the proximal region, while shorter fibers were found in the middle region [18]. Such inhomogeneity of the fiber lengths can alter the length–tension characteristics of the VI. In addition, the orientation of the muscle fiber with respect to the aponeurosis is different along the length of the aponeurosis. The pennation angle of VI has been shown to be smaller in the distal region than in the proximal and middle regions [18]. The different morphological properties of the central aponeurosis along the longitudinal axis may also contribute to its nonuniform displacement. The central aponeurosis is sheet-like as it courses proximally; however, it becomes rather flat in the mid-region and then oval near its broad insertion into the patella tendon [6,19]. Thus, the mechanical properties of VI are not entirely the same throughout the muscle. Additional mechanical characteristics would increase the stress transmitted by the more distal regions of the aponeurosis, which would result in higher strains. A greater aponeurosis cross-sectional area in the more distal regions is required to compensate for this behavior.

In addition to comparing the ensemble mean results, the local displacement of tissues at the voxel level (displacement distribution) is also important because it reflects the movement of fascicles and aponeuroses in detail. In particular, the displacements near the central aponeuroses observed in the present study might show the unique contributions of different muscles to create force and mechanical work transmission [20]. Thus far, it has been difficult to investigate this large displacement observed along the *Z*-axis direction near the central aponeurosis in the VI, as the VI is located deep inside the quadriceps muscles. Moreover, we found that the amount of *Z*-axis displacement near the central aponeurosis in VI was similar to the values of muscle tissues on the opposite side of the central aponeurosis (i.e., VL and VM). Therefore, the experimental observations in this study suggest that the different muscles in the quadriceps act synchronously, at least partly, to produce force and displacement along the direction of the line of action through the central aponeurosis. The central aponeurosis connects all the way to the patellar tendon, acting as one functional unit, although the quadriceps muscle consists of four muscles whose physiological and architectural properties are remarkably different, as discussed above.

**Figure 10:**
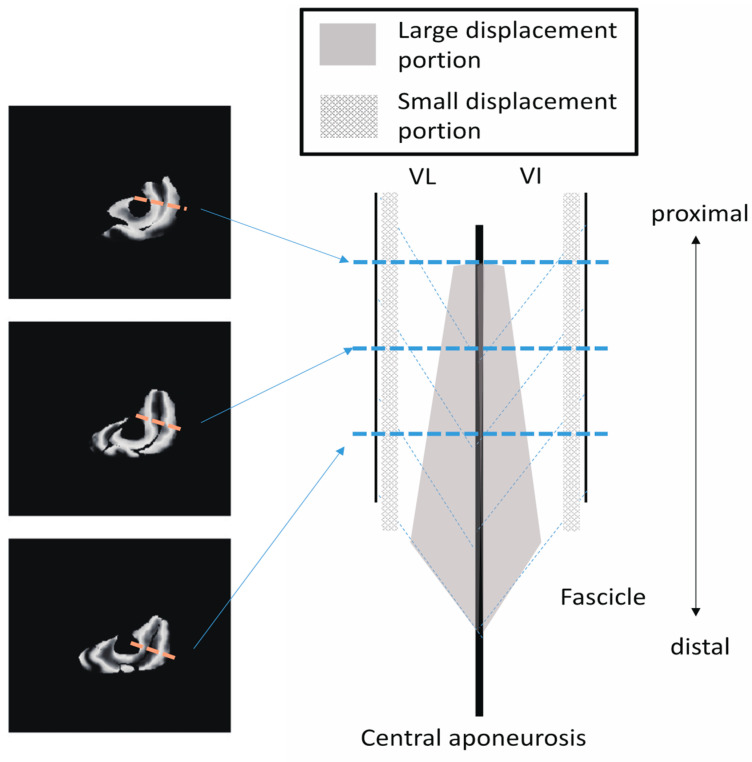
A schematic diagram on the sagittal cutting plane of the VI and VL portraying the differences in *Z*-axis displacement both in-plane and along the proximal–distal direction shown in Figure 8.

In addition to the amount of displacement, the areas with large *Z*-axis displacement are larger in the distal plane compared to the proximal plane, as shown in Figure 8a in red. This is a reflection of fascicle behavior, such as shortening and rotation of fascicles [21] during contractions. Moreover, the differences in the distribution of displacement along the proximal–distal direction imply heterogeneity of fascicle length changes along the fascicles. Figure 10 shows a schematic image of the VI and VL on a sagittal cutting plane portraying the differences in *Z*-axis displacement both in-plane and along the proximal–distal direction, as shown in Figure 8. From the data in the present study, the displacement of the distal fascicles near the central aponeurosis was larger than that of the proximal portion of the fascicles.

These results lead to the possibility that, even in the same fascicle, there is a division of roles, such as the exertion of force without a large length change, e.g., the proximal portion, or performing a length change without large force generation, e.g., the distal portion, depending on the location inside the muscles. In fact, such a division of roles has also been reported in the muscle–tendon complex, and it is believed that the muscle exerts force. However, the tendon tissue undergoes a change in length during jumping [22]. A similar division of roles may exist inside the muscle tissues. Heterogeneity in the spatial distribution of fascicle length changes during contraction has been reported in the medial gastrocnemius muscles using VEPC-MRI [15], but not in the quadriceps muscles. Heterogeneity in the aponeurosis strain has also been reported in the medial gastrocnemius muscle (Kinugasa et al. [10]). The length–velocity–force relationship [23] determines the force generation capacity of local contractile elements in the fascicles [24]. Thus, the local muscle tissue behavior during contraction as measured by VEPC-MRI yields important information for understanding muscle biomechanics in humans, such as force distribution and pressure distribution related to the deformation of tissues during contractions.

In the present study, the total displacement of not only VI but also VM was quite large, especially in the middle and distal portions. However, in terms of the *Z*-axis displacement, the VM exhibited a smaller displacement than VI. This difference between the total and *Z*-axis displacements in the VM was caused by large in-plane displacements, that is, large in-plane components in the lateral–medial and anterior–posterior (X and Y) directions. The ensemble average of the ratio of the Z-component displacement vector to the total displacement in the VM was less than 0.5, suggesting a larger displacement in the lateral–medial and anterior–posterior directions compared to that in other muscles. This movement would imply a different role of VM, for example, inward stability of the knee joint, in the four synergists. Further studies are required to clarify these differences and similarities.

The example of the distribution of the ratio of the Z-component displacement vector to the total displacement (Figure 9A) also indicates that the complex displacement of tissue changes three-dimensionally, with a combination of in-plane and out-of-plane displacements during contraction. An example of the displacement changes with time in all pixels of the middle section image (Figure 9B) shows that pixels with positive displacement are dominant. However, pixels with negative displacement are also present, which shows the opposite fascicle movement during contraction. It is difficult to measure such complicated movements of muscle tissues using other modalities (e.g., ultrasonic method). Future research using VEPC-MRI can reveal in detail the muscle contraction behavior.

The map of the vectors of the in-plane maximal displacement of the VL from the relaxation state (Figure 4) demonstrated great variations in the direction of displacement. The three-dimensional movements of muscle tissues under the skin during contractions affect the quality of data from many experimental methods, such as B-mode ultrasound and surface EMG. This is because the measurement device is fixed at one point on the surface of the skin. Thus, these data suggest that the imaging plane of ultrasound and the detection area of EMG can be changed by the movement of tissues during contraction. As large displacements may exceed the width of the ultrasound probe or the area of interest of the EMG electrodes, care should be taken.

## 5. Conclusions

We investigated the displacement of muscle tissues in sub-maximally contracting quadriceps using VEPC-MRI and a tracking algorithm. The results demonstrate the differences in the ensemble-averaged displacements of the synergist muscles. Different spatial distributions of the displacement patterns were observed in different muscles, including the VI muscles, which have not been explored in detail previously because of their deep position within the thigh. The results of the *Z*-axis displacement suggest that VI is the main generator of force and mechanical work, at least in terms of displacement in the four muscles. Additionally, the highest values of *Z*-axis displacement tended to concentrate, particularly, around the central aponeurosis, separating the VI from its surrounding muscles. Hence, the different muscles in the quadriceps act synchronously as one functional unit to produce force and displacement along the direction of the line of action through the central aponeurosis. The behavior is complex in each respective muscle, which possesses diverse physiological characteristics and architectures.

## Data Availability

The datasets generated and analyzed during the current study are available from the corresponding author upon reasonable request.
